# Impact of early enteral nutrition on ventilator associated pneumonia in intubated severe trauma patients: A propensity score-matched study

**DOI:** 10.3389/fnut.2023.1172526

**Published:** 2023-04-12

**Authors:** Su Wang, Xin Zhao, Qian Wang, Yongran Wu, Jiaxin Xu, Ruiting Li, Ting Zhou, Zheng Lv, Jihong Yang, Le Yang, Xiaojing Zou

**Affiliations:** ^1^Department of Critical Care Medicine, Union Hospital, Tongji Medical College, Huazhong University of Science and Technology, Wuhan, China; ^2^Department of Intensive Care Unit, Wuhan Third Hospital (Tongren Hospital of Wuhan University), Wuhan, China; ^3^Department of Critical Care Medicine, People’s Hospital of Chongyang County, Xianning, China; ^4^Department of Emergency Medicine, Tongji Hospital, Tongji Medical College, Huazhong University of Science and Technology, Wuhan, China

**Keywords:** early enteral nutrition, ventilator-associated pneumonia, severe trauma patients, invasive mechanical ventilation, propensity score matching (PSM)

## Abstract

**Background:**

Early enteral nutrition (EN) is recommended for critically ill patients. However, the impact of early EN on intubated severe trauma patients remains unclear.

**Methods:**

Severely traumatized adult patients who received invasive mechanical ventilation (MV) for more than 48 h during intensive care unit (ICU) stay at our institution between 2017 and 2022 were retrospectively included. Early EN was defined as EN initiation ≤48 h from ICU admission and late EN >48 h. Propensity score matching (PSM) analysis was used to compare outcomes between the groups. The primary endpoint was the incidence of ventilator-associated pneumonia (VAP). Multivariable logistic regression analysis was performed to identify independent predictors of delayed EN.

**Results:**

For final analysis, 337 intubated severe trauma patients were available, including 204 (60.5%) in the early EN group and 133 (39.5%) in the late EN group. After PSM, early EN patients had a lower incidence of VAP (12.9 vs. 25.8%, *p* = 0.026) and a shorter length of hospital stay (21 vs. 24 days, *p* = 0.015) compared to late EN patients. There was no demonstrable difference in mortality between the two groups. Abdominal trauma, massive blood transfusion, and serum albumin were identified as independent risk factors for delayed EN.

**Conclusion:**

Early EN decreased the VAP rate and reduced the length of hospital stay in invasively ventilated patients with severe trauma. Abdominal injury, massive blood transfusion and low albumin were associated with delayed EN.

## Introduction

Severely injured trauma patients who require ventilator support and intensive care unit (ICU) admission are at risk of nosocomial pneumonia development (1). Nosocomial infections in the ICU, particularly ventilator-associated pneumonia (VAP), pose a considerable economic burden globally, as patients often need prolonged mechanical ventilation (MV) and have significantly longer stays in the ICU and hospital (2). VAP occurred more frequently in intubated trauma patients than in other critically ill patients, and contributed to increased morbidity and mortality (3, 4). It has been reported that the incidence of VAP can reach up to 60.6% among severe trauma patients (5). How to effectively prevent VAP in trauma patients remains an important issue.

The responses following the traumatic injuries such as muscle breakdown, immune suppression, and cytokine cascade potentially deteriorate the nutritional status, which predisposes the patients to infectious complications (6). Nutritional support including enteral nutrition (EN) and parenteral nutrition is a crucial factor in the fight against hospital pneumonia (7). However, the reports on the relationship between EN and the development of VAP seem to be conflicting. Some authors have indicated that early EN was associated with increased VAP risk (8, 9), whereas others have observed the opposite (10). To date, in severely traumatized patients, the impact of early EN on VAP is also unclear. Moreover, although early initiation of EN is recommended in the trauma patients, the evidence is also inconsistent as to whether it provides a survival benefit (11, 12).

Here, our primary purpose was to compare the effects of early vs. late EN on VAP occurrence and other clinical outcomes in invasively ventilated patients with severe trauma using a propensity score model. The second purpose was to identify factors that were associated with delayed EN.

## Materials and methods

### Ethical statement

This study adhered to the Declaration of Helsinki and was approved by the Ethical Committee of the Union Hospital (No. 0167-1), Tongji Medical College, Huazhong University of Science and Technology, Wuhan, China. Due to the retrospective nature of the study, patient informed consent was waived.

### Study population

We retrospectively reviewed the medical records of all trauma patients consecutively admitted to the ICU of our institution between January 2017 and December 2022. In this study, the patients were enrolled if they met the following inclusion criteria. (i) Who was an adult patient (≥18 years old). (ii) Who had an injury severity score (ISS) ≥ 16 points (13). (iii) Who received EN. (iv) Who underwent invasive MV for more than 48 h. (v) Who were not confirmed with COVID-19. Excluded patients had been treated with tracheostomy before ICU arrival; had already received invasive MV for at least 12 h before ICU admission; switched to palliative care; had incomplete medical records and missing key data.

### Data collection and definitions

We extracted patient data from electronic medical record system in our institution. The parameters were collected in the present study including gender, age, body mass index (BMI), history of smoking, Charlson Comorbidity Index (CCI), initial Glasgow Coma Scale (GCS), brain trauma, abdominal trauma, chest trauma, spinal trauma, pelvic trauma, ICU admission diagnosis of shock and trauma-induced acute respiratory distress syndrome (ARDS), massive blood transfusion, ISS, sequential organ failure assessment (SOFA) and acute physiology and chronic health evaluation II (APACHE II) within 24 h after ICU arrival, serum creatinine, total bilirubin and albumin at ICU admission, and the time to delivery of EN after admission to the ICU.

The primary outcome of our study was the incidence of VAP. Other outcome variables were the duration of invasive MV, the length of ICU and hospital stay, and 28-day mortality.

Body mass index was defined as body weight (kg)/body height (m^2^). Massive blood transfusion was defined as adults transfused packed red blood cells ≥10 units within 24 h or >4 units within 1 h (14). Early EN was defined as EN initiation within 48 h after ICU admission and late or delayed EN as initiation after 48 h of ICU admission, based on the recommendation of nutrition support therapy guidelines in the adult critically ill patients (15). EN was initiated according to the assessment of the physician in charge at the time. Patients were routinely given EN by a nasogastric feeding tube at our institution, and the gastric tube was considered to be exchanged for a jejunal tube when gastric motility disorder occurred and no improvement was observed after administration of metoclopramide. VAP was defined as a pneumonia arising >48 h after endotracheal intubation. VAP diagnoses were documented by the new onset of fever or leukocytosis and purulent sputum, with new lung infiltrate, and the positive microbiological sample of endotracheal aspirate (≥10^5^ colony-forming units [cfu]/mL) or bronchoalveolar lavage (≥10^4^ cfu/mL) or protected brush specimens (≥10^3^ cfu/mL) (16).

### Statistical analysis

All statistical analyses were performed using IBM SPSS software (version 26.0), and differences were considered statistically significant at *p* < 0.05 in this study. Normal distribution was checked by Kolmogorov–Smirnov test for all continuous variables. Categorical variables were presented as frequency (percentage) and non-normally distributed continuous variables as medians and interquartile ranges. To compare baseline characteristics between the two groups, chi-square test or Fishers exact test was used for categorical variables, and Mann-Whitney non-parametric test was used for continuous variables with non-normal distributions.

To minimize the influence of potential confounders, a propensity score matching (PSM) analysis was conducted. The covariates used for propensity matching were the twenty variables presented in Table 1. To create a matched sample, nearest neighbor matching was performed with a caliper width of 0.1 on the propensity-score with a matching ratio of 1:1. A subgroup analysis was also conducted, and we evaluated the treatment-by-covariate interactions to explore potential heterogeneity of the treatment effects relative to the sites of trauma. In this study, the primary outcomes stratified by trauma sites were compared in the PSM cohort and the *p*-values for the interactions were examined using the Cochran-Mantel-Haenszel test in subgroups. In order to identify the independent risk factors for delayed EN, factors with a *p*-value of less than 0.05 in the univariable analysis were entered into the multivariable logistic regression analysis with a forward stepwise procedure.

**TABLE 1:** Baseline characteristics of the severe trauma patients receiving invasive mechanical ventilation.

Characteristics	Early EN (*n* = 204)	Late EN (*n* = 133)	*p*-value
**Demographics**			
Gender^§^			0.447
Male	157 (77.0)	107 (80.5)	
Female	47 (23.0)	26 (19.5)	
Age, years^§^			0.102
≥70	23 (11.3)	8 (6.0)	
<70	181 (88.7)	125 (94.0)	
Body mass index, kg/m^2#^	22.9 (20.8–25.2)	23.0 (21.3–25.0)	0.367
Smoking history^§^	56 (27.5)	39 (29.3)	0.709
Charlson comorbidity index, points^§^			0.123
0	162 (79.4)	117 (88.0)	
1	33 (16.2)	14 (10.5)	
≥2	9 (4.4)	2 (1.5)	
Initial GCS, points^#^	15 (15–15)	15 (15–15)	0.171
**Site of injury**			
Brain^§^	101 (49.5)	45 (33.8)	0.005*
Abdominal^§^	21 (10.3)	56 (42.1)	<0.001*
Chest^§^	112 (54.9)	68 (51.1)	0.497
Spinal^§^	110 (53.9)	49 (36.8)	0.002*
Pelvic^§^	39 (19.1)	33 (24.8)	0.213
**ICU admission diagnosis**			
Shock^§^	85 (41.7)	82 (61.7)	<0.001*
Trauma-induced ARDS^§^	116 (56.9)	73 (54.9)	0.721
**Within 24 h after ICU admission**			
Massive blood transfusion^§^	24 (11.8)	39 (29.3)	<0.001*
ISS^#^	42 (27–75)	43 (27–75)	0.499
SOFA^#^	9 (8–12)	10 (8–12)	0.376
APACHE II^#^	15 (11–20)	16 (11–22)	0.220
**Laboratory test at ICU admission**			
Serum creatinine, μmol/L^#^	67.4 (54.0–87.2)	75.8 (58.8–108.7)	0.004*
Total bilirubin, μmol/L^#^	16.7 (11.8–23.9)	14.6 (11.2–21.9)	0.114
Albumin, g/L^#^	26.5 (22.8–30.6)	23.4 (17.4–27.6)	<0.001*

EN, enteral nutrition; ICU, intensive care unit; GCS, Glasgow coma scale; ARDS, acute respiratory distress syndrome; ISS, injury severity score; SOFA, sequential organ failure assessment; APACHE, acute physiology and chronic health evaluation.

^§^ Categorical variables are presented as frequencies with percentages.

^#^Non-normally distributed continuous variables are presented as medians with interquartile ranges.

*p < 0.05.

## Results

### Population characteristics

**FIGURE 1:**
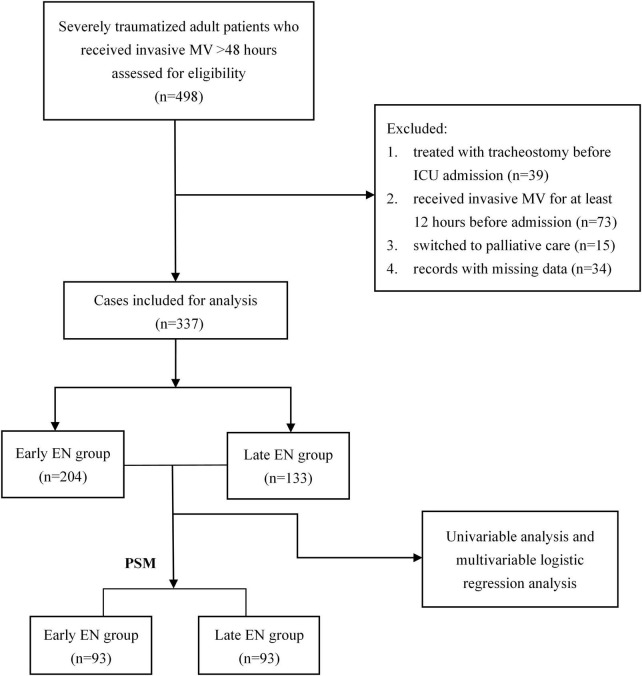
Flow chart of the study. ICU, intensive care unit; EN, enteral nutrition; MV, mechanical ventilation; PSM, propensity score matching.

A total of 498 mechanically ventilated patients with severe trauma met the inclusion criteria. Among them, 39 patients had been treated with tracheostomy before ICU admission, 73 patients had already received invasive MV for at least 12 h before admission, 15 patients switched to palliative care, and 34 patients had incomplete data records (Figure 1). After excluding the cases based on the exclusion criteria, the remaining 337 cases were further analyzed in this study. Of these patients, 78.3% were male, 9.2% were older than 70 years, 28.2% had a smoking history, 17.2% had a CCI score ≥1, 43.3% suffered a brain injury, 22.8% suffered an abdominal injury, 53.4% suffered a chest injury, 47.2% suffered a spinal injury, 21.4% suffered a pelvic injury, 49.6% occurred the shock, 56.1% occurred the trauma-induced ARDS, 18.7% received massive blood transfusion. The median BMI of the entire study population was 22.9 kg/m^2^, the median GCS score was 15 points, the median ISS score was 42 points, the median SOFA score was 10 points, the median APACHE II score was 15 points, the median serum creatinine value was 70.0 μmol/L, the median total bilirubin value was 16.0 μmol/L, and the median albumin value was 25.7 g/L.

There were 204 patients (60.5%) delivered with early EN, while 133 patients (39.5%) with late EN. Compared with the late EN group, the early EN group had a lower serum creatinine value (67.4 vs. 75.8 μmol/L, *p* = 0.004), a higher albumin value (26.5 vs. 23.4 g/L, *p* < 0.001), a higher proportion of brain injury (49.5 vs. 33.8%, *p* = 0.005) and spinal injury (53.9 vs. 36.8, *p* = 0.002), a lower proportion of abdominal injury (10.3 vs. 42.1%, *p* < 0.001), shock (41.7 vs. 61.7%, *p* < 0.001) and massive blood transfusion (11.8 vs. 29.3%, *p* < 0.001). The detailed comparison was presented in the Table 1.

### Early EN and patient outcomes

In invasively ventilated patients with severe trauma, we assessed the impact of early EN on the primary outcome of VAP development. The overall incidence of VAP was 21.4% (72/337), and the early EN patients had a lower incidence (16.7 vs. 28.6%, *p* = 0.009, Table 2) compared to the late EN patients. A significantly shorter length of hospital stay was also observed in the early EN group (21 vs. 26 days, *p* < 0.001). But no significant effect of early EN on invasive MV time, length of stay in ICU and all-cause mortality rate was demonstrated (Table 2). The 28-day mortality rate for the entire population was 6.2%.

**TABLE 2:** Outcomes in early and late enteral nutrition groups.

Variables	All (*n* = 337)	Early EN (*n* = 204)	Late EN (*n* = 133)	*p*-value
Ventilator-associated pneumonia^§^	72 (21.4)	34 (16.7)	38 (28.6)	0.009*
Invasive mechanical ventilation, days^#^	6 (3–10.5)	6 (3–10)	6 (4–13)	0.273
ICU stay, days^#^	9 (5–16)	8 (5–15)	10 (5–17)	0.201
Hospital stay, days^#^	23 (16–35)	21 (15–31)	26 (18.5–44)	<0.001*
28-day mortality^§^	21 (6.2)	12 (5.9)	9 (6.8)	0.743

EN, enteral nutrition; ICU, intensive care unit.

^§^ Categorical variables are presented as frequencies with percentages.

^#^Non-normally distributed continuous variables are presented as medians with interquartile ranges.

*p < 0.05.

**FIGURE 2:**
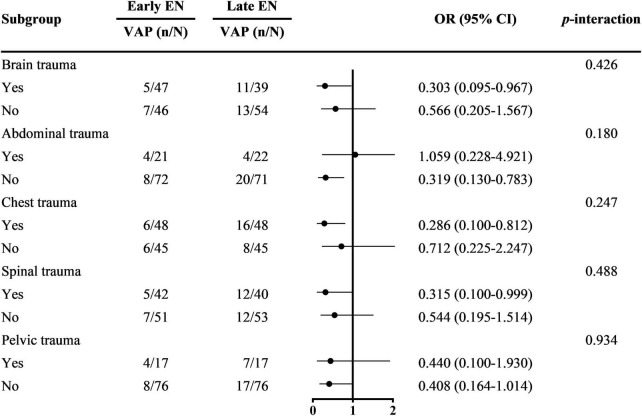
Treatment effect on VAP by subgroup. VAP, ventilator-associated pneumonia; EN, enteral nutrition; OR, odds ratio; CI, confidence interval.

To further investigate the role of early EN delivery in preventing VAP and improving prognosis in severely traumatized population, we performed PSM to balance clinical factors between the two groups. PSM yielded a cohort of 93 patients in the early EN group and 93 patients in the late EN group. After PSM, between-group differences for all variables were eliminated (Table 3). The significant results between the groups remained similar after matching by propensity score (Table 4), that is, patients with delayed EN had higher VAP rates and longer hospital stays. In the early EN group, shorter invasive ventilator duration, shorter ICU stay and lower mortality were also observed, but no significant differences were present. We did a subgroup analysis, and subgroup results were presented as a forest plot (Figure 2). There were no significant interactions between treatment groups and different sites of trauma in regard to the occurrence of VAP (*p*-interaction >0.05). The effects of early EN on the primary outcome were similar regardless of trauma sites.

**TABLE 3:** Comparison of baseline characteristics in early and late enteral nutrition groups after PSM.

Characteristics	Early EN (*n* = 93)	Late EN (*n* = 93)	*p*-value
**Demographics**			
Gender			0.111
Male^§^	68 (73.1)	77 (82.8)	
Female^§^	25 (26.9)	16 (17.2)	
Age, years^§^			0.601
≥70	9 (9.7)	7 (7.5)	
<70	84 (90.3)	86 (92.5)	
Body mass index, kg/m^2#^	22.5 (20.8–24.4)	22.9 (21.0–24.8)	0.338
Smoking history^§^	25 (26.9)	28 (30.1)	0.626
Charlson comorbidity index, points^§^			0.539
0	77 (82.8)	78 (83.9)	
1	11 (11.8)	13 (14.0)	
≥ 2	5 (5.4)	2 (2.2)	
Initial GCS, points^#^	15 (15–15)	15 (15–15)	0.719
**Site of injury**			
Brain^§^	47 (50.5)	39 (41.9)	0.239
Abdominal^§^	21 (22.6)	22 (23.7)	0.862
Chest^§^	48 (51.6)	48 (51.6)	1.000
Spinal^§^	42 (45.2)	40 (43.0)	0.768
Pelvic^§^	17 (18.3)	17 (18.3)	1.000
**ICU admission diagnosis**			
Shock^§^	48 (51.6)	53 (57.0)	0.462
Trauma-induced ARDS^§^	46 (49.5)	50 (53.8)	0.557
**Within 24 h after ICU admission**			
Massive blood transfusion^§^	19 (20.4)	24 (25.8)	0.385
ISS^#^	42 (26–75)	43 (27–75)	0.388
SOFA^#^	10 (8–12)	10 (8–12)	0.789
APACHE II^#^	15 (11–21)	15 (11–20)	0.542
**Laboratory test at ICU admission**			
Serum creatinine, μmol/L^#^	67.9 (55.8–87.9)	74.6 (58.8–105.9)	0.182
Total bilirubin, μmol/L^#^	14.9 (11.2–21.8)	16.0 (11.2–23.6)	0.996
Albumin, g/L^#^	25.2 (19.7–29.4)	24.6 (19.6–28.6)	0.524

PSM, propensity score matching; EN, enteral nutrition; ICU, intensive care unit; GCS, Glasgow coma scale; ARDS, acute respiratory distress syndrome; ISS, injury severity score; SOFA, sequential organ failure assessment; APACHE, acute physiology and chronic health evaluation.

^§^ Categorical variables are presented as frequencies with percentages.

^#^Non-normally distributed continuous variables are presented as medians with interquartile ranges.

**TABLE 4:** Comparison of outcomes in early and late enteral nutrition groups after PSM.

Variables	Early EN *n* = 93	Late EN *n* = 93	*p*-value
Ventilator-associated pneumonia^§^	12 (12.9)	24 (25.8)	0.026*
Invasive mechanical ventilation, days^#^	5 (3–10)	6 (3–14)	0.648
ICU stay, days^#^	8 (5–15.5)	10 (5–18)	0.265
Hospital stay, days^#^	21 (13.5–32)	24 (18–40.5)	0.015*
28-day mortality^§^	6 (6.5)	7 (7.5)	0.774

PSM, propensity score matching; EN, enteral nutrition; ICU, intensive care unit.

^§^ Categorical variables are presented as frequencies with percentages.

^#^Non-normally distributed continuous variables are presented as medians with interquartile ranges.

*p < 0.05.

### Multivariable model for delayed EN

A multivariable logistic regression analysis was carried out to identify the independent risk factors for delayed EN. Seven statistically significant variables in the univariable analysis were included for the further multivariable regression analysis. The results showed that the variables independently associated with delayed EN were abdominal trauma [odds ratio (OR): 6.087; 95% confidence interval (CI): 3.384, 10.949; *p* < 0.001], massive blood transfusion (OR: 2.657; 95% CI: 1.409, 5.010; *p* = 0.003), and serum albumin (OR: 0.953; 95% CI: 0.918, 0.988; *p* = 0.010; Table 5).

**TABLE 5:** Multivariable analysis of independent risk factors for delayed enteral nutrition.

Characteristic	Coefficient	Standard error	OR (95% CI)	*p*-value
Abdominal trauma	1.806	0.300	6.087 (3.384–10.949)	<0.001*
Massive blood transfusion	0.977	0.324	2.657 (1.409–5.010)	0.003*
Albumin	−0.049	0.019	0.953 (0.918–0.988)	0.010*

CI, confidence interval; OR, odds ratio.

*p < 0.05.

## Discussion

Multiple studies have shown that the burden of VAP in trauma patients is significant (17, 18). The observed incidence rate of VAP among the severely traumatized patients in the present study was within the range (11.9–28.3%) previously reported (19, 20). As VAP is known to result in escalating the cost of care and increasing length of stay and mortality, studies have explored the effect of some treatment strategies on reducing its prevalence, such as use of probiotic preparation (20). Nutritional intervention is also considered to be an alternative means to influence the prognosis of trauma patients. Over the past two decades, there has been a growing body of clinical studies surrounding the application of EN to reduce the risk of VAP (21). Regrettably, the impact of early EN support on VAP development in the trauma population has not been well recognized yet.

To our knowledge, this is the first study with a propensity score-matching method to report superiority for early EN over late EN in reducing VAP occurrence in invasively ventilated patients with severe trauma. Our findings also indicated that early EN delivery exerted no significant effect on mortality of these patients, but significantly decreased the length of hospital stay. The risk factors for delayed EN were further analyzed in this study, and the multivariable analysis revealed that abdominal trauma, massive blood transfusion on the first day after ICU admission and lower albumin prevented early delivery of EN.

In this study, even after PSM, provision of early EN to the severe trauma patients significantly decreased the VAP rate. A study based on a nationwide inpatient database found that early EN application in patients with severe traumatic brain injury reduced nosocomial pneumonia (22). Similar effect was observed in acute pancreatitis patients, and the nutritional status of patients was also improved (23). Distinctive and complex metabolic changes after trauma make severe trauma patients more susceptible for developing malnutrition (6, 24). One review further indicated that depending on the severity of injury, energy expenditure increased by 20–50% in trauma patients compared with patients after elective surgery (6). However, numerous studies have revealed that malnutrition in hospitalized patients was related to the increased complications and mortality and prolonged hospital stays (25, 26). In addition to providing protein and calories for critically ill trauma patients, EN also produces non-nutritional benefits. Early enteral feeding following trauma can prevent intestinal mucosa atrophy which can occur rapidly after injury (27). Studies have indicated that early EN maintains the functional gut integrity and prevents bacterial translocation, thereby reducing the risk of systemic infections (28, 29). Moreover, it has been found that stress hyperglycemia mediated by stress-induced insulin resistance following severe trauma is associated with both infection and death in critically injured patients (30), whereas enteral feeding modulates metabolic responses, attenuating the insulin resistance (31). Therefore, early EN application might help reduce the risk of infections.

Enteral nutrition could elicit immunologic changes, and immunologic communication between the gastrointestinal (GI) tract and mucosal surfaces throughout the body might exist *via* a common mucosal immunity (32). The transfer of immune cells between the gut and the lungs might play a beneficial role in enhancing the capacity of the host to fight infection (33). In addition, the transport of soluble microbial components and metabolites *via* the circulation is one means of communication between the gut microbiota and the lungs (33). Samuelson et al. also suggested that the GI microbiota played a crucial role in immune adaptation and initiation at the distal mucosal sites, such as the lung (34). Moreover, studies have indicated that lack of enteral feeding could lead to loss of established anti-viral and anti-bacterial defenses within the respiratory tract, and impaired ability to cope with new infectious challenges (35). Although it was pointed out that nasogastric tube EN increased the risk of reflux and aspiration of gastric contents and reduced the prevention of VAP (36), our findings supported the benefits of early EN application in the severely traumatized population. Besides, Croce et al. demonstrated that use of the ventilator bundle could not work well to prevent VAP in trauma patients (37). Hence, the early delivery of EN may be a useful strategy.

Despite EN delivery being an important part of standard care for critically ill patients, it has been documented that only one third of patients have EN initiated within the 48-h recommendation (38). Approximately 21.0% of patients receiving delayed EN in the medical ICU was reported (39). Moreover, a higher proportion (64%) of patients with severe traumatic brain injury had delayed EN (22), compared with our study population. It is obvious that patient factors may influence the success of early EN delivery.

As a complex injury within a complex cavity, abdominal trauma was a potent risk factor for delayed EN in our study, with an adjusted OR of 6. Yin et al. expressed that their hesitation to introduce early EN in abdominal trauma patients was due to fear of feeding intolerance, although EN could be delivered at 12 h after operation or 24 h after injury in these patients (40). Perhaps our result could also be explained by this. Their study, however, showed that early EN did not increase feeding intolerance and was related to reduced infectious complications (40). In addition, abdominal viscera exposure has led to EN withheld by some clinicians in patients with open abdomen due to the concerns about paralytic ileus and subsequent aspiration and bowel dilation risk (41). Nonetheless, Dissanaike et al. have suggested that early EN appears safe and is associated with the reduction in pneumonia in this patient population (42). Unless contraindicated, late initiation of enteral feeding may be unwise.

Another independent risk factor of delaying EN was massive blood transfusion. Hemorrhage is the most common cause of early death in trauma patients, and massive blood transfusion is required to resuscitate traumatically injured patients with complex physiologic derangements (43). Physiologically, shock shunts blood flow away from the GI tract, however, gut perfusion remains depressed in spite of effective shock resuscitation (44). Thus, there is a concern that EN in shock further harms the already impaired splanchnic perfusion (12), which may partially explain the relatively high proportion of patients with massive blood transfusion receiving late EN. However, one review indicated that early EN in patients with shock was well tolerated and was related to preserved splanchnic blood flow and improved clinical outcomes (45). Moreover, previous study has demonstrated that massive transfusion is independently associated with VAP among critically ill trauma patients (46). Torrance et al. noted that the transfusion of blood products enhanced immunosuppressive inflammatory response caused by polytrauma with resultant increased susceptibility to nosocomial infection (47). Therefore, we speculate that providing early EN support to trauma patients with massive transfusions may have potential benefits.

Critical illness is characterized by inflammation, and hepatic reprioritization of protein synthesis results in lower serum concentrations of albumin (48). Similarly, a low overall albumin level occurred in severe trauma population of this study. According to our results, the lower the albumin value, the more likely it is to delay EN application. This may be interpreted by the consideration of hypoalbuminemia inducing delayed gastric emptying and abnormal intestinal peristalsis (49), while enteral feeding may increase the risk for regurgitation, pulmonary aspiration, and eventual VAP when GI dysfunction is present (50).

This work was constrained by several limitations. First, it was a retrospective study with its inherent biases, and was performed in a single-center setting without a large sample size. Second, the method of PSM was used for this study, however, we could not control for the balance of all potential confounding variables. A prospective study with a large number of patients is required. Third, the different routes for EN support may influence the outcomes (51). Fourth, due to the limited sample size, subgroup analyses may have been underpowered. Last, VAP is difficult to diagnose. The prevalence of VAP varies according to the different diagnostic criteria, and its use affects the time to diagnosis and associated mortality rate (52).

## Conclusion

The present study of early vs. late EN showed that early EN decreased the risk of VAP developing and reduced the duration of hospital stay in invasively ventilated patients with severe trauma. Abdominal injury, massive blood transfusion and lower albumin level were associated with delayed EN. Despite applying PSM to adjust for bias in the current study, randomized controlled trial is crucial to further corroborate the results.

## Data availability statement

The original contributions presented in this study are included in the article/supplementary material, further inquiries can be directed to the corresponding authors.

## Ethics statement

The studies involving human participants were reviewed and approved by the Ethics Committee of the Union Hospital (No. 0167-1), Tongji Medical College, Huazhong University of Science and Technology, Wuhan, China. Written informed consent for participation was not required for this study in accordance with the national legislation and the institutional requirements.

## Author contributions

XjZ, LY, and SW: conceptualization. XjZ and LY: supervision. RL, TZ, and ZL: investigation. QW, YW, JX, ZL, and JY: data curation. SW, XZ, QW, and YW: methodology. SW, XZ, and JX: writing–original draft preparation. XjZ, LY, RL, and TZ: writing–review and editing. All authors contributed to the article and approved the submitted version.
